# Supplementary material for: A Human 3D neural assembloid model for SARS-CoV-2 infection
Source: Res Sq. 2021 Feb 12:rs.3.rs-214352. Preprint. [Version 1] doi: 10.21203/rs.3.rs-214352/v1 (PMC7885926; doi:10.21203/rs.3.rs-214352/v1)
Supplement: Supplement [file 1e5ebed5759bb54ed1d146b1.docx]

**Supplementary Information**

**Human 3D neuronal ‘assembloids’ model SARS-CoV-2 infection**

Lu Wang^1, 2^, David Sievert^1, 2^, Alex E. Clark^3^, Hannah Federman^4^, Benjamin D. Gastfriend^5^, Eric Shusta^5^, Sean P. Palecek^5^, Aaron F. Carlin^3*^, Joseph Gleeson^1, 2, 6, 7*^

**Extended data Figs. 1-7**

Extended Data Fig.1 PLCs show appreciable ACE2 expression level.

Extended Data Fig.2 PCCOs retain COs primary cellular composition and structure.

Extended Data Fig.3 Characterization of PLCs within PCCOs.

Extended Data Fig.4 PCCOs promote neural differentiation.

Extended Data Fig.5 PCCOs show increased neuron-glial interaction.

Extended Data Fig.6 PCCOs show robust SARS-CoV-2 infection absent in COs.

Extended Data Fig.7 COs show little evidence of SARS-CoV-2 infection.

**Supplementary Information**

Table 1. List of primer sequences used for qPCR.

**Supplementary Datasets**

Supplementary Dataset 1. List of differentially expressed proteins in PCCO vs. CO (TMT4-MS), fractions of scRNA-seq results, related to Fig.1 and Extended Data Figs. 2-4.

Supplementary Dataset 2. Raw statistical data for the study, related to Figs.1-2 and Extended Data Figs. 1-2, and 7.

Supplementary Dataset 3. List of differentially expressed genes in PCCOs and COs upon SARS-CoV-2 infection, related to Figs.1-2 and Extended Data Fig. 5.

**Supplementary notes**

Methods in details

**Report Summary**

**Editorial Policy Checklist**

**Extended data Fig.1 PLCs show appreciable ACE2 expression level.**

**
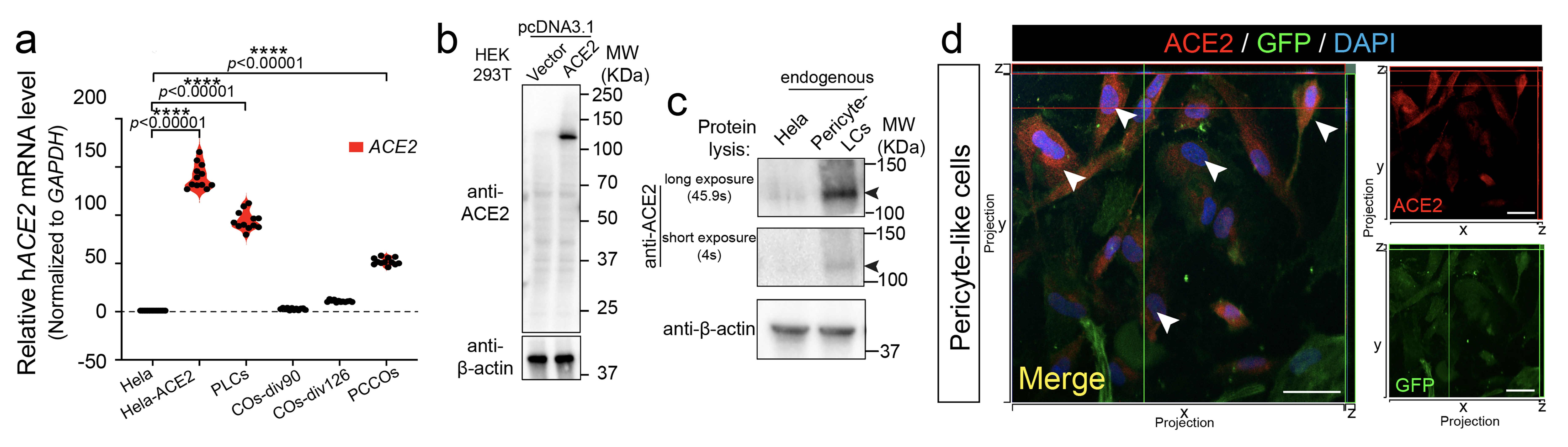
**

**a.** RT-qPCR shows elevated mRNA expression level of *ACE2* in PLCs. *GAPDH*: reference control, Hela-ACE2 stable cell line used as positive control, Hela cells used as negative control. n=12 includes 3 independent biological replicates and 4 technical replicates for each of the biological replicate. Multiple t-test was used to calculate significance followed by a Sidak multiple-comparison test correction. **** p<0.00001. **b-c.** Western Blot shows ACE2 expression level in PLCs. β-actin used as loading control. Overexpression of ACE2 in HEK293T cells was used to qualify the antibody (**c**). Hela cells used as negative control. Arrows: ACE2 band. **d.** Immunostaining shows ACE2 expression in PLCs. PLCs were harvested for immunostaining against ACE2, shown in red. PLCs labeled with GFP in green, blue: DAPI, bar: 20 µm. Confocal xz/yz projections used to show colocalization.

**Extended Data Fig. 2 PCCOs retain COs primary cellular composition and structure**

**
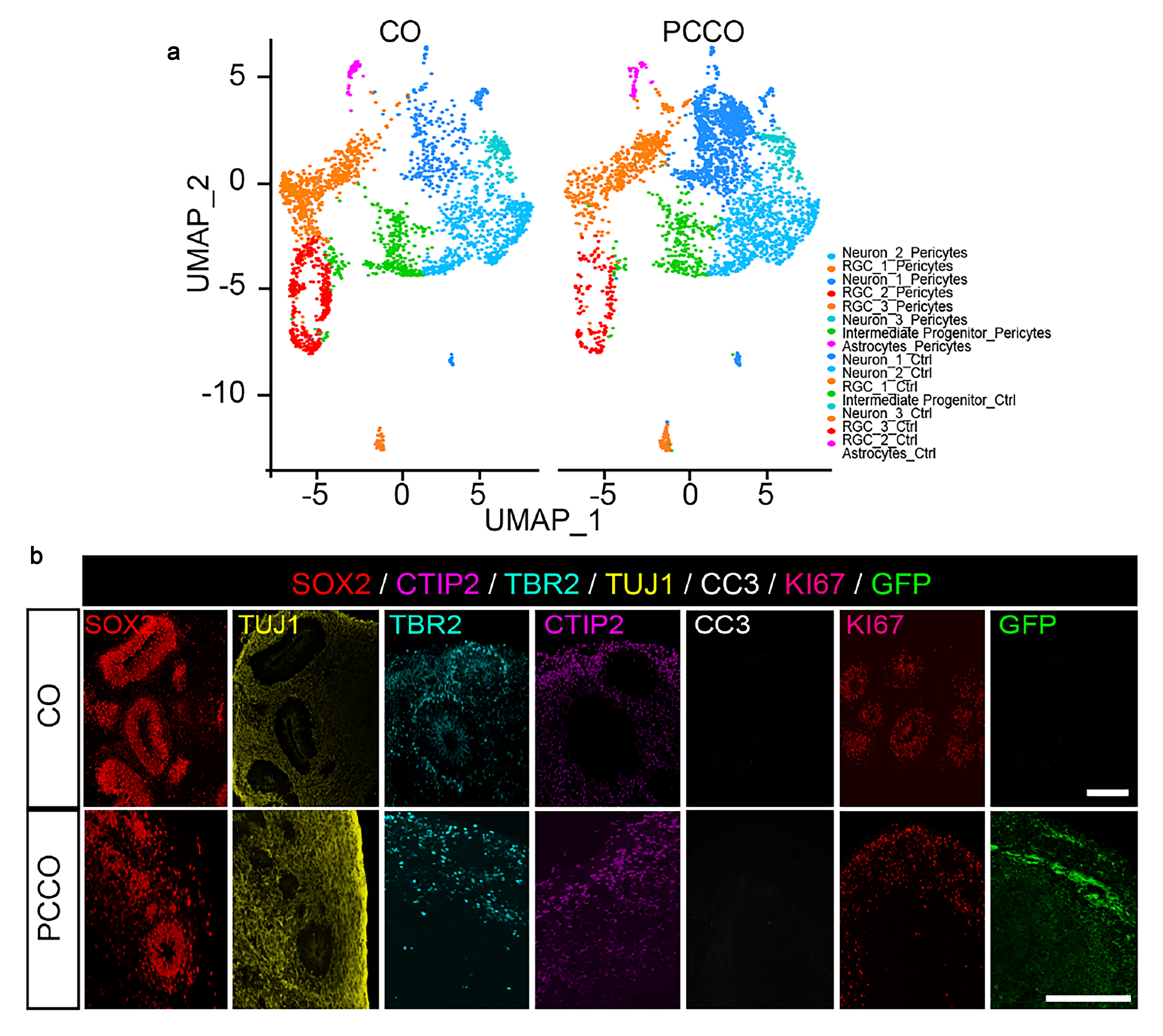
**

**a.** Split UMAPs show the cell compositions in CO and PCCO. **b.** Immunostaining against SOX2 (red), CTIP2 (magenta), TBR2 (cyan), TUJ1 (yellow), CC3 (gray) and KI67 (chocolate red) on both CO and PCCO sections. GFP shows GFP^+^ PLCs present in PCCO. Tissue from column 1-4 and columns 5-6 are serial sections. Bars: 400µm.

**Extended Data Fig.3 Characterization of PLCs within PCCOs.**

**
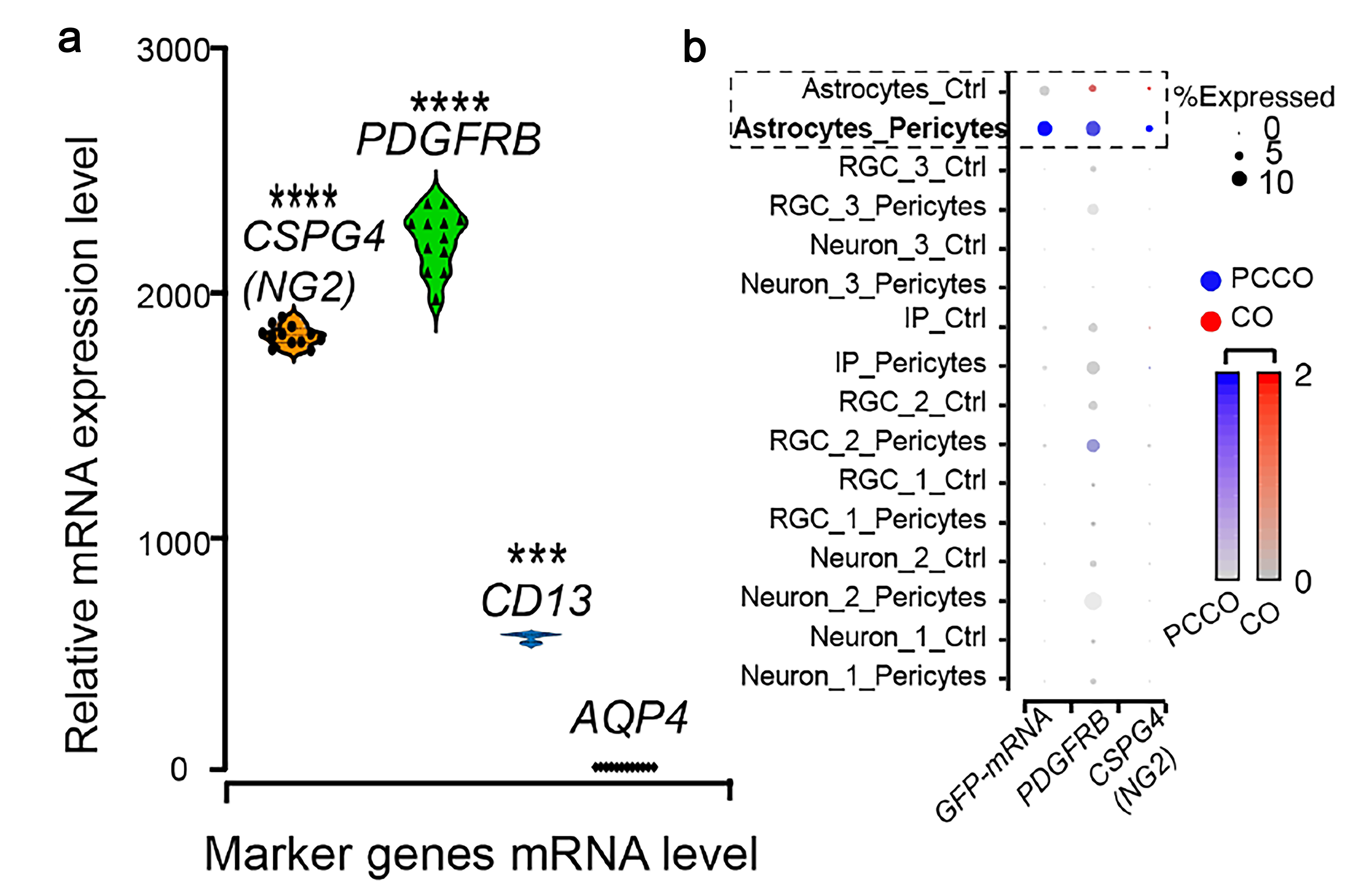
**

**a.** GFP^+^ PLCs within PCCOs show high mRNA expression of pericyte marker genes (*NG2, PDGFRB, CD13*). GFP^+^ PLCs were harvested for RNA extraction and subsequent RT-qPCR against *CSPG4* (*NG2), PDGFRB, CD13* and *AQP4. GAPDH* was used as reference control. *AQP4* used as sample control. n=12 includes 3 independent biological replicates and 4 technical replicates for each biological replicate. Multiple t-test was used to calculate significance followed by a Sidak multiple-comparison test correction. **** p<0.00001. **b.** Dot-plot shows *GFP-mRNA* expressed in astrocytes cluster in PCCO together with *PDGFRB* and *CSPG4 (NG2).* Dot size: % cells demonstrating expression of gene of interest. Dot shade: average expression for gene of interest in log-fold scale. For example, *GFP* is expressed only in PCCOs (blue dot vs. gray dot) whereas *PDGFRB* is expressed in both but in a much higher percent of cells in PCCOs.

**
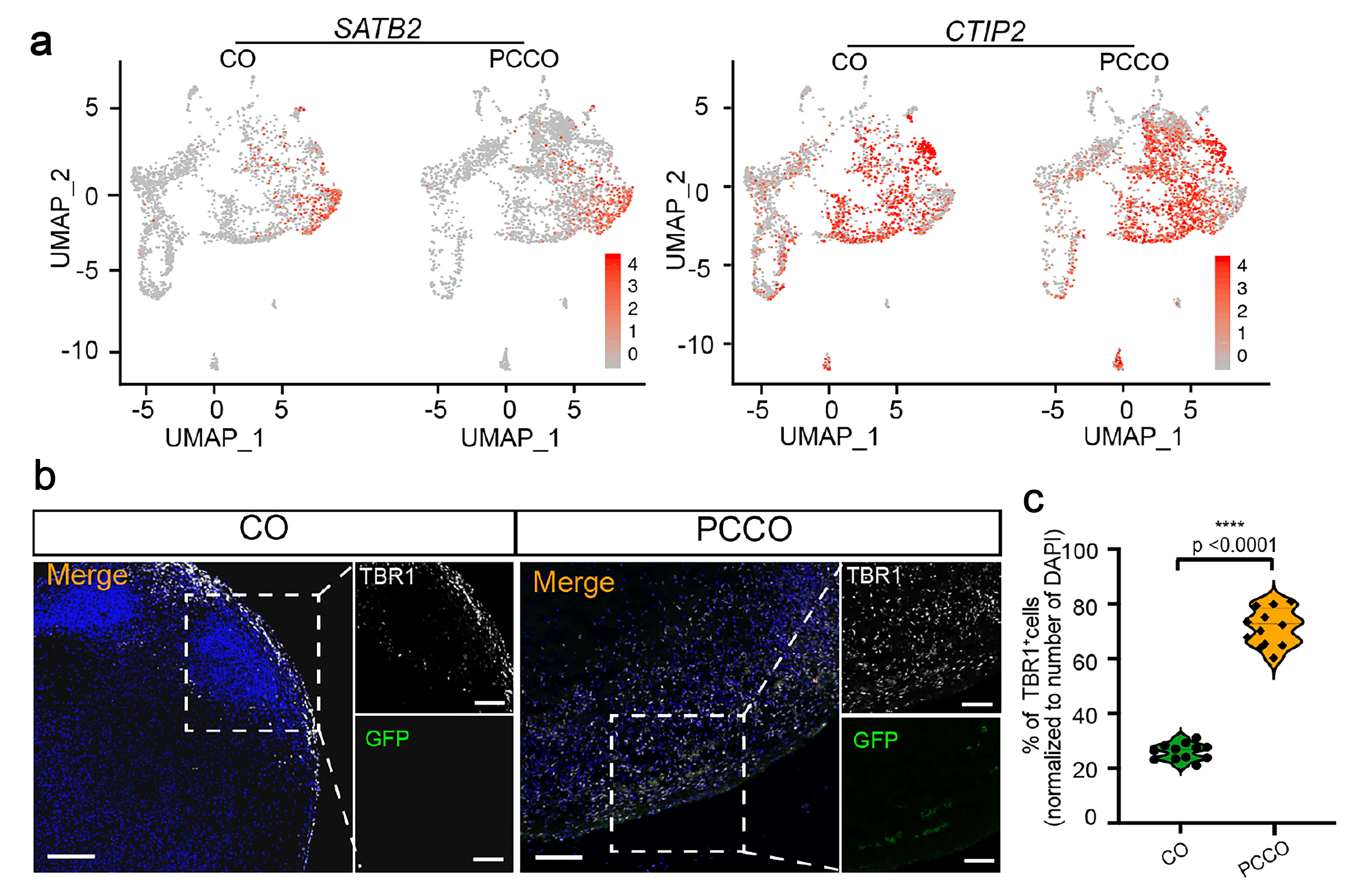
Extended Data Fig.4 PCCOs promote neural differentiation.**

**a.** Split UMAPs show *SATB2* and *CTIP2* expression in different cell populations of CO and PCCO. **b.** Immunostaining shows TBR1 expression in COs and PCCOs. Organoids were harvested for immunostaining against anti-TBR1, shown in gray. PLCs labeled with GFP in green, blue: DAPI, bar: 400 µm. Zoom in bar: 100 µm. **c.** Quantification of TBR1^+^ cells ratio in D. n=12 includes 3 independent biological replicates from different COs/PCCOs and 4 different image regions from at least 3 different section regions in D. Multiple t-test was used to determine the significance followed by a Sidak multiple-comparison test correction. **** p<0.00001.

**Extended data Fig.5 PCCOs show increased neuron-glial interaction.**

**
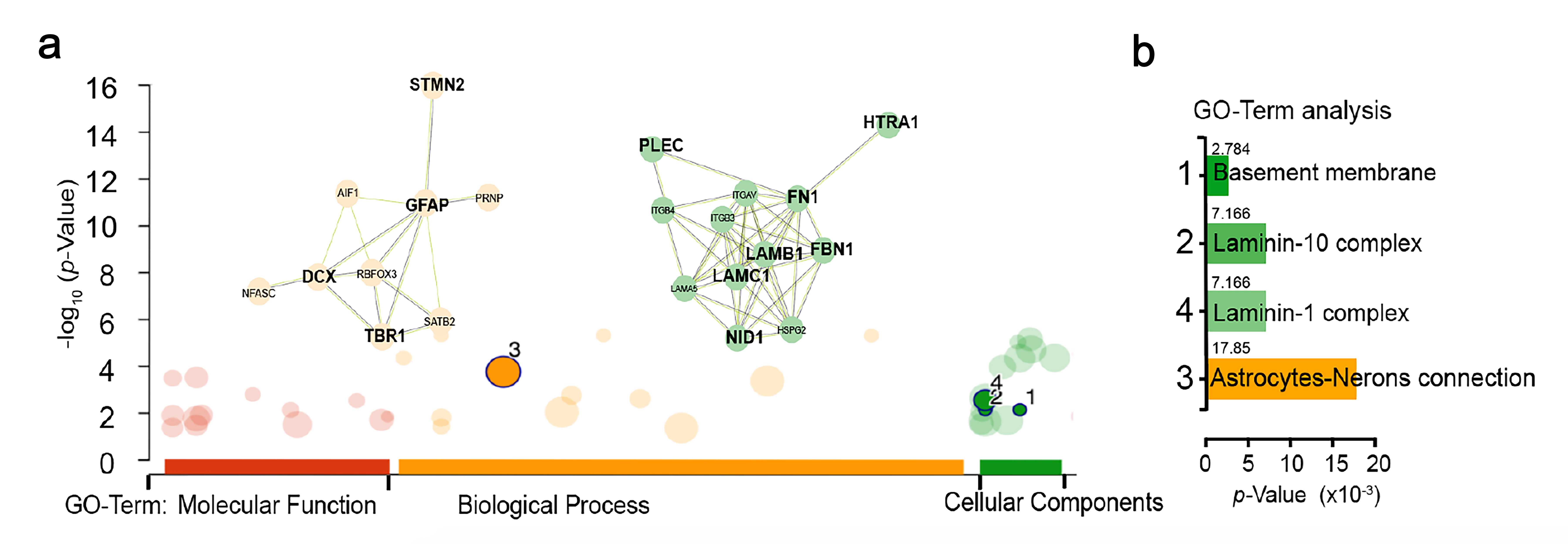
**

**a.** PCCOs and COs were labeled with TMT4 isobaric tags then subjected to LC-MS, to identify differential protein abundance based upon differential peptides analysis. GFAP-DCX-TBR1-STMN2, and Laminin-complex interaction matrixes were highlighted in PCCO compared to CO, based upon go-profile and string analysis to highlight the interaction modules. *y-*axis -log10 *p-*value. **b**. GO-Term analysis indicated enriched expression of Basement membrane and Astrocytes-Neuron interaction in the TMT4-LC-MS experiment. *x*-axis: p-value x10^-3^.

**Extended Data Fig.6 PCCOs show robust SARS-CoV-2 infection absent in COs.**


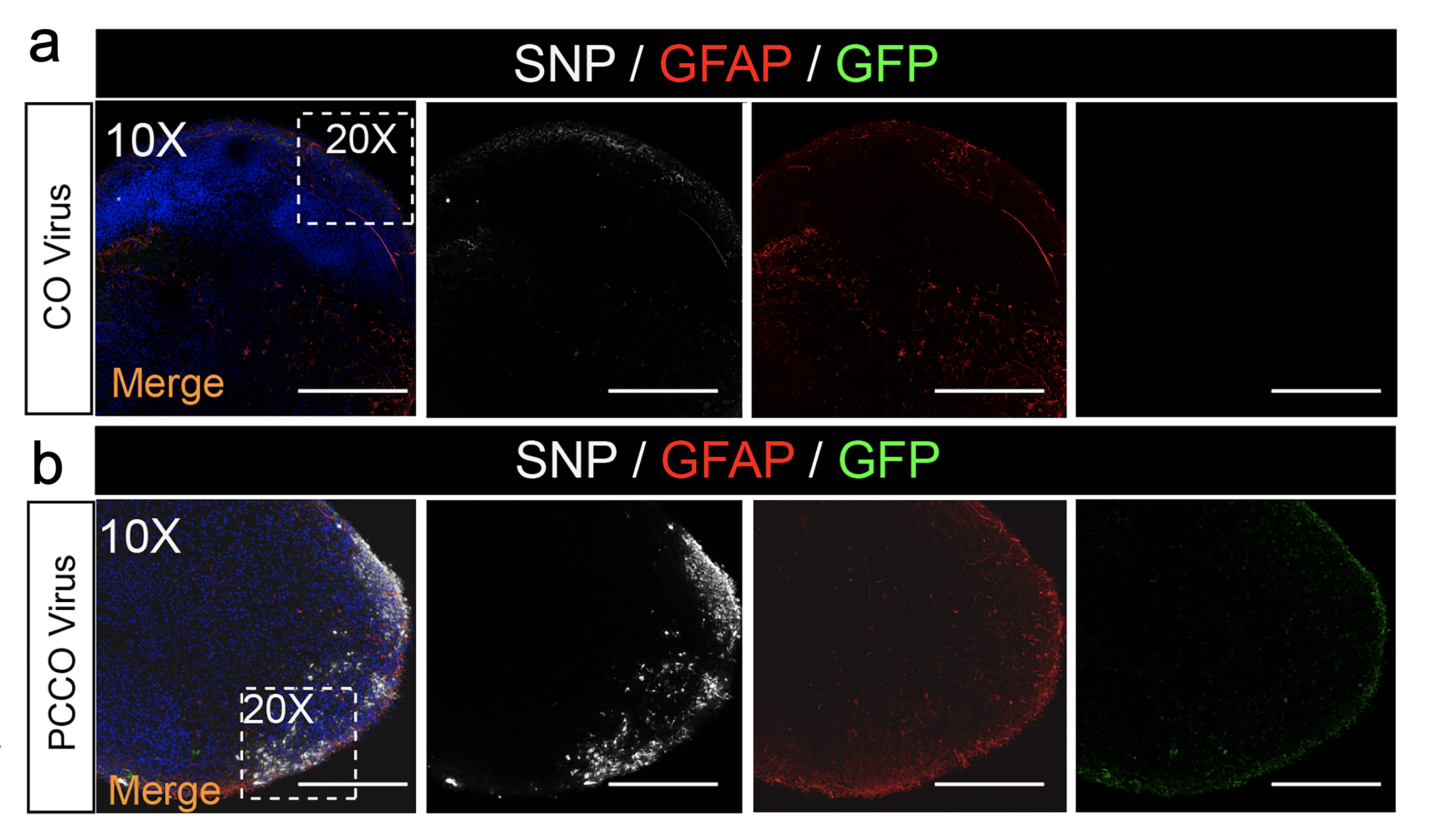


**a-b.** SNP and GFAP staining shows few SNP^+^ cells in COs (**a**) and robust SNP^+^ cells in PCCOs (**b**) 72h after SARS-CoV-2 virus exposure. Bar: 400µm. DAPI: blue, GFP: PLCs, GFAP: astrocytes, and SNP: SARS-CoV-2-NP. The dashed white boxes show the 20x region shown in **Fig. 3b-e**. Bar: 400µm.

**Extended Data Fig.7 COs show little evidence of SARS-CoV-2 infection.**

**
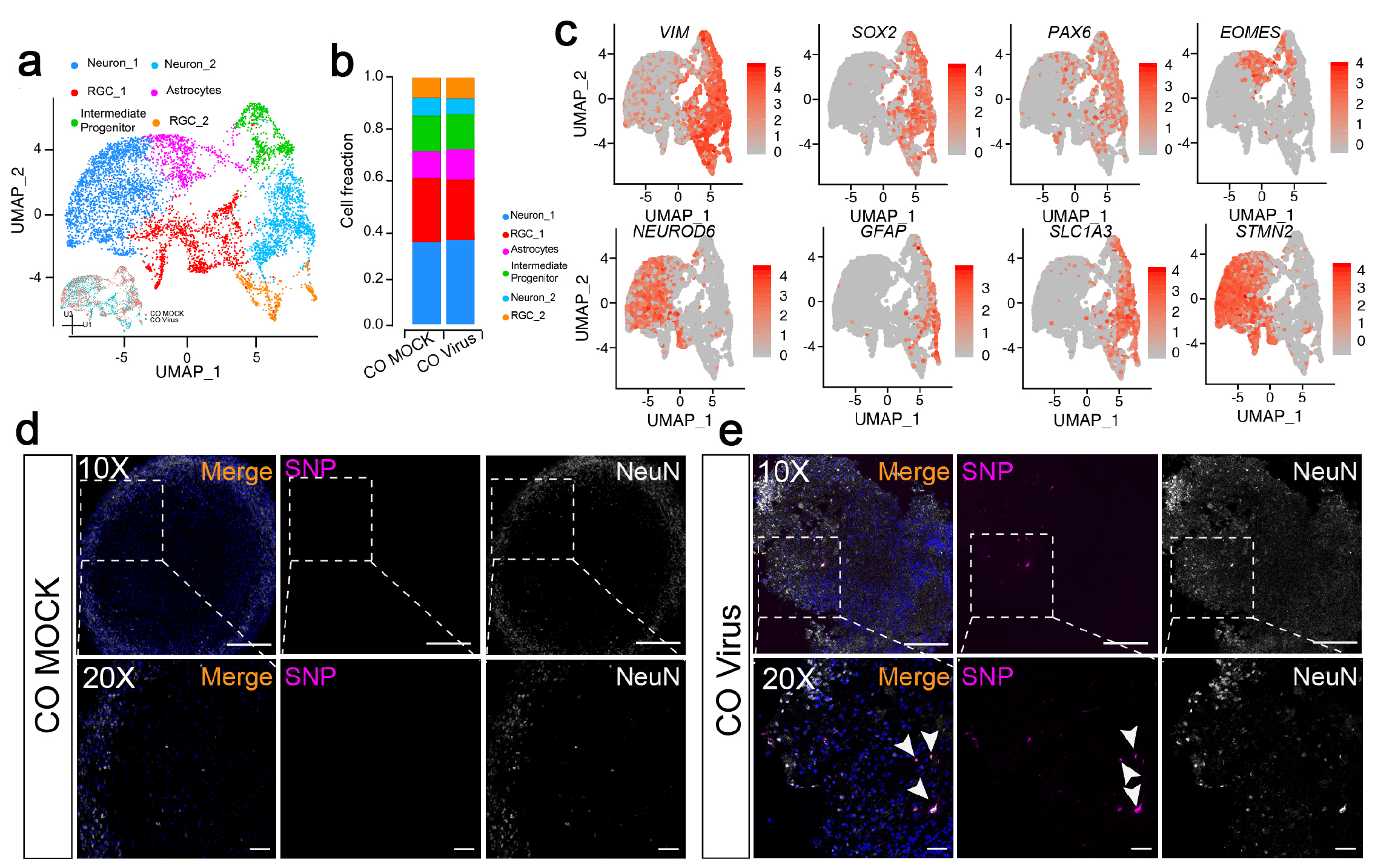
**

**a.** Merged UMAP shows the cell compositions in COs with/without SARS-CoV-2 virus exposure. **b.** Cell fractions show unchanged cellular composition in COs before and after virus exposure. **c.** UMAPs show expression features in different cell populations. **d.** SARS-CoV-2-NP (SNP) and NeuN staining in CO sections with or without SARS-CoV-2 virus exposure. DAPI: blue, bar: 400µm for 10X, 100μm for 20x.

**Supplementary Information**

Table 1. List of primer sequences used for qPCR.

| Genes (human) | Forward sequencing (5’-3’) | Reverse sequencing (5’-3’) |
| --- | --- | --- |
| *PDGFRB* | GTCATCCATCAACGTCTCTG | CGGATGTGGTAAGGCATATC |
| *NG2* | CAGGATGGCTTCCACTTTC | CTGGACCTCGTACTCAATCT |
| *CD13* | CCACTGACGTCATCATCATC | TCGCTGTCCATCTCATACT |
| *AQP4* | CCTTTGGACCTGCAGTTATC | ATCAGGTCATCCGTCTCTAC |
| *ISG15* | GGCAGCGAACTCATCTTT | CCGATCTTCTGGGTGATCT |
| *ACE2* | GGTCTTCTGTCACCCGATTT | CATCCACCTCCACTTCTCTAAC |
| *Transcripts (SARS-CoV2)* | Forward sequencing (5’-3’) | Reverse sequencing (5’-3’) |
| *SARS-CoV-2-S1* | GACCCCAAAATCAGCGAAAT | TCTGGTTACTGCCAGTTGAATCTG |
| *SARS-CoV-2-S2* | TTACAAACATTGGCCGCAAA | GCGCGACATTCCGAAGAA |
| *SARS-CoV-2-S3* | TCATCACGTAGTCGCAACAG | CAAAGCAAGAGCAGCATCAC |
| *RNP* | AGATTTGGACCTGCGAGCG | GAGCGGCTGTCTCCACAAGT |

**Supplementary notes**

**Methods**

**Human Induced Pluripotent Stem Cells, Neural Crest Stem Cells Culture**

HEK293T cells (sex typed as female), Hela and H1 ESC (sex typed as male) were obtained from ATCC (CRL-11268^TM^), ATCC (ATCC^®^CCL-2^TM^) and WiCell (WAe001-A), and were not further authenticated. Generation of neural crest stem cells (NCSCs) was described previously ^1^. Human induced pluripotent stem cells (hiPSCs) were from CIRM (CIRM-IT1-06611). All cells were regularly mycoplasma negative. HEK 293T and HELA cells were maintained in Dulbecco’s modified Eagle’s medium (DMEM, Gibco) with 10% FBS (Gibco), 1X GlutaMax (Gibco), 1X NEAA (Gibco), 1X Sodium Pyruvate solution (Gibco), 100U/ml Penicillin/Streptomycin (Gibco). H1 cells and hiPSCs were maintained in culture with mTeSR^TM^1 culture medium (STEMCELL Technologies) on Matrigel coated dishes (Corning). NCSCs were maintained in E6 medium (STEMCELL Technologies) with 10ng/ml FGF2 (PEPROTECH), 22.5µg/ml heparin sulfate (Sigma), 10uM SB431542 (R&D), 1µM CHIR99021 (Selleckchem) and 1µM dorsomorphin (Selleckchem) in laboratory of Dr. Eric Shusta at Wisconsin ^1^. In brief, NCSCs were derived from human pluripotent stem cells (hPSCs) with E6 basal medium in addition of 1μM CHIR99021, 10μM SB431542, 10μg/L FGF2, and 1μM dorsomorphin for 14 days. At day15, cells were labeled with NCSC microbeads (Miltenyi Biotec; conjugated with antibody against the NCSCs markers HNK1 and p75-NGFR) to perform magnetic activated cell sorting (MASC) sorting to obtain a pure population (99.8%) of HNK1^+^/p75-NGFR^+^ NCSCs. pLV-EF1a-EGFP-Puro construct was obtained from Dr. Fred Gage’s lab as a gift. Lentiviral packaging plasmids pMD2.G, pPAX2 were obtained from Addgene. pcDNA3.1-hACE2 plasmid was obtained from Dr. Tom Rogers at Scripps as a gift.

**Lentivirus packaging and PLCs generation**

PLCs were derived from hPSC-derived NCSCs with 9 days of culture with E6 medium (STEMCELL Technologies) with 10% FBS ^1^. GFP lentiviral particles were generated by co-transfection of HEK293T cells with pLV-EF1a-EGFP-Puro and pMD2.G, psPAX2 using lipo2000. Supernatant containing viral particles was harvested after 72h, 0.22µm filtered, and lentiX (Clontech) concentrated. 10µl GFP-lentiviral particles was used to infect PLCs at NCSCs differentiation day 7, after 48h infection 10 µM puromycin was used to select GFP^+^ PLCs. After 7 days selection, GFP^+^ PLCs were maintained in E6 medium with 10% FBS for another two days for further use.

**Human cortical brain organoid (CO) and pericyte containing cortical organoid (PCCO) culture**

H1 and hiPSCs were maintained in mTeSR and passaged according to manufacturer’s recommendations. Cortical brain organoids were generated as previously described ^2,3^. H1 cells at 36 passages and hiPSC at 12 passages were dissociated into single cells with Accutase. In total, around 9000 cells were then plated in each well of an ultra-low-attachment 96-well plate (Corning, CLS3474) in “Cortical differentiation medium” with 20 µM Rock inhibitor (Selleckchem), 5 µM SB431542 (R&D), 3 µM endo-IWR-1 (Selleckchem) for the first 4d, then cultured for an additional 13d in cortical differentiation medium with 5 µM SB431542, 3 µM endo-IWR-1, with medium changes every 2d. On 18d, the immature organoids were transferred into ultra-low-attachment 6-well plate and cultured for another 16d in “Organoid differentiation medium.” Media was changed every 3–4d. On 35d, media was changed to “Maturation medium” supplemented with 1% Matrix High Concentration (HC), Growth Factor Reduced (GFR) Matrigel (Corning), with medium replaced every 3–4d for additional 34d. HC-GFR Matrigel was added fresh at each change. On 70d, media was changed to “Long-term maintenance medium” supplemented with 2% HC-GFR-Matrigel, 50 ng/ml BDNF (R&D, 248-BD-025/CF) and 50 ng/ml GDNF (R&D, 212-GD-010/CF) for long term organoid maintenance. “Cortical differentiation medium”: Glasgow’s MEM (GMEM), 20%Knock-Out Serum Replacement (KO-SR), 1X Non-Essential Amino Acid (NEAA), 1X Sodium Pyruvate, 1X β-Mercaptoethanol, 1X Pen/Strep. “Organoid differentiation medium”: DMEM/F12, 1X N2, 1X NEAA, 1X CD-lipid concentrator, 1XPen/Strep. “Maturation medium”: DMEM/F12, 10%FBS, 1X N2, 1X CD-lipid concentrator, 1X Pen/Strep, 1%HC-GFR Matrigel, 30μg/ml Heparin. “Long term maintenance medium”: DMEM/F12, 20%FBS, 1X N2, 1X B27w/o vitamin A, 1X CD-lipid concentrator, 1X Pen/Strep, 2% HC-GFR Matrigel, 30μg/ml Heparin, 50ng/ml BDNF, 50ng/ml GDNF, 50ng/ml NT3.

For PCCO generation, at CO 60 div, 2x10^5^ GFP+ PLCs were integrated into each of the CO in a low attachment 96-well plate. PLC integrated COs were maintained in Maturation medium for 14 days.

**SARS-CoV-2 infection of pericyte-like cells, COs, and PCCOs**

All work with SARS-CoV-2 was conducted in Biosafety Level-3 conditions at the University of California San Diego following the guidelines approved by the Institutional Biosafety Committee. SARS-CoV-2 isolate USA-WA1/2020 (BEI Resources) was propagated and infectious units quantified by plaque assay using Vero E6 (ATCC) cells. For PLC infection, 2x10^4^ PLCs were cultured on a coverslip as 2D in low attachment 24-well plate. SARS-CoV-2 was added at a MOI of 0.5 for one hour in 1ml of culture medium with rocking every 10-15 minutes. After one hour, the supernatant was removed, cells were washed once with media and 1ml of fresh media was added. The cells were incubated for 48h at 37°C, 5% CO2. After 48hrs, 1ml of fresh medium was added and the cells were cultured for another 24h. For infection of COs and PCCOs, SARS-CoV-2 in 1ml culture medium at an approximate MOI of 0.5 was added and cells incubated for 48h at 37°C, 5% CO2. After 48hrs, 1ml fresh medium was added into the culture for another 24h. To calculate the MOI for CO and PCCO, counts from previous single cell dissociations^2^ were used to determine the cell number in the organoids. The cell numbers were approximately 1~1.5M cells per CO and approximately 1.2~1.7M cells per PCCO.

**Detection of viral mRNA and replication using RT-qPCR**

For viral RNA quantification, PLCs, COs or PCCOs were washed twice with PBS and lysed in TRIzol (ThermoFisher). RNA was extracted using the Qiagen-RNA extraction Kit (Qiagen). 2µg RNA was used to generate cDNA with SuperScript III First-Strand Synthesis Kit (Invitrogen). 20ng cDNA was used to perform qPCR with iTaq Universal SYBR Green Supermix and the CDC-N1/N2/N3-SARS-CoV-2 primers mix (IDT) at a final concentration of 100nM for each primer using a Bio-Rad Real-Time PCR system. Ribnucleoprotein (RNP) was used as reference. To determine viral replication, RNA was extracted from 200µl of inoculated PLC culture medium at 24h, 48h and 72h post-virus infection by QIAmp Viral RNA mini-kit (Qiagen). Sample Cq values were converted to viral copies per reaction using the standard curve’s linear regression model. Values in dot plots represent fold changes first normalized to *RNP* and then normalized to MOCK infection for each individual biological sample. All the qPCR primers were listed in Table S1 and raw data is available in the Table S5.

**Plaque assay**

Viral supernatants were 10-fold serially diluted in DMEM without serum. Vero E6 cells in 12-well plates were washed with PBS, and 200uL of virus dilution was added per well and incubated 1 h at 37°C with rocking every 10-15 min. The inoculum was removed and 1mL of overlay (0.6% agarose in MEM with 4% FBS) was added to each well. Overlays were prepared by mixing equal volumes of 1.2% agarose and 2xMEM supplemented with 8% FBS, 2x L-glutamine, 2x non-essential amino acids, and 2x sodium bicarbonate. Assays were incubated 48 h at 37°C and fixed by adding 2mL 10% formaldehyde/well for at least 24 h. Overlays were removed and monolayers were stained with 0.025% crystal violet in 2% EtOH.

**Immunostaining and imaging for CO and PCCO**COs and PCCOs were fixed in 4% paraformaldehyde for 72h before removal from BSL3. The fixed COs and PCCOs were washed with PBST (PBS with 0.25% Tween 20) three times for 5 min, allowed to sink in 30% sucrose at least overnight at 4 °C, embedded in 15%/15% gelatin/sucrose solution and sectioned at 20 µm. 30min antigen retrieval (Tri-sodium citrate, 0.1% Tween-20, pH 6.0) was performed on the CO/PCCO sections followed by 20min 0.5% Triton X-100 permeabilization at room temperature. The sections were then blocked with 5% BSA in PBS for 1 h at room temperature. After washing with PBST 3 times for 5 min, sections were incubated with primary antibodies in 5% BSA/Triton X-100 in PBS at the following dilutions: SOX2 (R&D, AF2018-SP, 1:100), TUJ1 (Biolegend, 801202, 1:1000), Cleaved Caspase 3 (Cell Signaling Technology, 9661S, 1:500), Ki67 (BD-Biosciences, 550609, 1:1000), CTIP2 (Abcam, ab28448, 1:500), TBR2 (Abcam, EPR19012, 1:250), GFAP (Abcam, ab4674, 1:250), TBR1 (Abcam, ab183032, 1:250), LAMB1 (Abcam, ab44941, 1:100), SARS-CoV2-Nucleocapsid (SinoBiological, AB_2827977, 1:500), NeuN (Abcam, ab177487, 1:100), p53 (Abcam, ab90363, 1:300), PDGFR-*β* (R&D, AF1042, 1:100), αSMA (Invitrogen, 14-9760-82, 1:200), NG2 (ThermoFisher Scientific, PA5-92029, 1:100), ACE2 (R&D, AF933, 1:100) overnight at 4 °C, washed three times with PBST for 10 min, then incubated with secondary antibodies (Alexa Fluor^TM^ 488 donkey anti-mouse lgG (H + L), 1915874, 1:1000; Alexa Fluor^TM^ 594 donkey anti-rabbit lgG (H + L), 1890862, 1:1000, Alexa Fluor^TM^ 594 donkey anti-chicken lgG (H + L),703585155, 1:1000, Alexa Fluor^TM^ 594 donkey anti-rat lgG (H + L), 712585153, 1:1000, Alexa Fluor^TM^ 594 donkey anti-mouse lgG (H + L), 715585150, 1:1000, Alexa Fluor^TM^ 647 donkey anti-mouse lgG (H + L), 715605151, 1:1000, Alexa Fluor^TM^ 647 donkey anti-rabbit lgG (H + L), 711605152, 1:1000) together with DAPI (ThermoFisher Scientific, D1306, 1:50000) for 2 h at room temperature, washed with PBST three times for 5 min, and mounted with Fluoromount-G® (Southern Biotech, 0100-01). All the images were taken with ZEISS LSM880 Airyscan, with post-acquisition analysis done in ImageJ-6.

**Light Sheet Imaging of PCCO**

PCCOs were harvested for clearing in PBST in a 1.5ml EP tube (1PCCO/tube). CUBIC ^4^ was used for clearing. In brief, PCCOs were sunk into 1.5ml R1 (25% urea, 25% Quadrol, 15% Triton-X-100)/PBST solution at RT overnight. Solution was then changed to 1.5ml fresh R1, and incubated at RT for at least 24h until PCCOs turned clear. After 24h, R1 solution was replaced with 1.5ml R2 solution (25% urea, 50% sucrose, 10% triethanolamine) along with DAPI (1:1000) for incubation overnight at RT, then embedded into 1% Agarose solution for imaging. A 5X lens was used for imaging with a light sheet microscope (Zeiss Z1) according to manufacturer recommendations. The images were further developed with IMARIS (Oxford Instruments).

**CO/PCCO dissociation and dead cell removal**

COs and PCCOs were dissociated using AccuMax (STEMCELL Technology) with 10µM Rock inhibitor. COs/PCCOs were washed with PBS once, before adding 500μl AccuMax, for trituration. CO/PCCO pieces were then incubated at 37^o^C for 20min, with gentle trituration at 10min during dissociation. After 20min, 3ml Neuronal Medium was added into the dissociation system, and gentle trituration was performed to obtain single cell suspension. The cell suspension was centrifuged at 500g, RT for 10min, supernatant removed, and cells re-suspended with 100µl of Neuronal Medium. To remove the dead cells, 1µl Dead Cell Removal cocktail (Annexin V, STEMCELL Technology) was added into the dissociated CO/PCCO single cells suspension together with 1µl Biotin selection cocktail (Annexin V, STEMCELL Technology), gently mixed and incubated at RT for 3min. After 3min incubation, RapidSpheres^TM^ (STEMCELL Technology) was vortexed for 30s at RT and 2µl added to the above mixture. At the same time, 850µl Neuronal Medium was added and gently pipetted 2~3 times. Tubes were then placed into a magnetic holder and incubated for 3min at RT. After incubation, the supernatant was collected in a new tube for 10X GEM generation.

**Single cell library preparation and sequencing**

10X sc-RNA-seq-3’-V3.1 kit (10X Genomics) was used to generate the GEM, cDNA and library were generated according to the manufacturer’s instructions (10X Genomics). Briefly, live cells were partitioned into nanoliter-scale Gel Bead-In-Emulsions (GEMs) with the 10x Chromium Controller (10X Genomics), 1000 cells were targeted. Upon cell lysis and dissolution of the Single Cell 3′-V3.1 Gel Bead within the droplet, primers containing an Illumina P7 and R2 sequence, a 14 bp 10XBarcode, a 10 bp randomer, and a poly-dT primer sequence were released and mixed with the cell lysates and bead-derived Master Mix. Barcoded, full-length cDNA from poly-adenylated mRNA was then generated in each individual bead, then individual droplets were broken and homogenized before the remaining non-cDNA components were removed with silane magnetic beads (Invitrogen). The libraries were then size-selected, and the R2, P5 and P7 sequences were added to each selected cDNA during end repair and adapter ligation. After Illumina bridge amplification of cDNA, each library was sequenced using the Novaseq6000 with PE150bp at the IGM Core in UCSD, around 20M reads were requested for each sample.

**CO/PCCO TMT4 quantitative Protein Mass Spectrometry**

Three COs and three PCCOs were harvested into 1.5ml cold PBS, then centrifuged at 1500rpm at 4°C for 10min. After centrifugation, all PBS was removed and the CO/PCCO samples were flash frozen in liquid N2. The frozen cell pellets were analyzed by TMT4 quantitative mass spectrometry at the UCSD Proteomics Core.

**Data processing of single-cell RNA-seq and Mass Spectrometry**

Single cell RNA-seq sequencing were demultiplexed into Fastq files using the Cell Ranger (10x Genomics, 4.0) mkfastq function. Samples were then aligned to GRChg38-2020 10x genome reference. The count matrix was generated using the count function with default settings. SARS-CoV-2 (USA-WA1/2020) genome and GFP-CDS were written into GRChg38-2020 human genome reference as a gene with mkref function in cell ranger 4.0. Seurat package (v.3.1.5) in RStudio (with R v. 3.5.3), used for the downstream analysis. Features expressed in less than 5 cells, cells with less than 300 unique features, or high mitochondrial content over 5% were discarded. scTransform function was used to wrap the technical variation; FindIntergrationAnchors and IntegrateData functions were used to integrate all libraries metrics into a single matrix. Principal Component Analysis (PCA) together with the PCEIbowPlot function were used to determine the inflection point. Clusters were determined using Runheatmap, FindNeighbors and Findclusters function within Seurat. Cells were considered infected if they carried the transcripts aligned to SARS-CoV-2 viral genome. Differentially expressed genes from the FindMarkers function were used to perform DAVID-GO-Term analysis over representation tests for both up-regulated and downregulated genes in each condition shown in (Figure. 2r, Supplementary Datasets 1-2).

Mass Spectrometry data were generated and analyzed by the proteomic core at UCSD (Table S2). The protein with significance over 15 were used for GO-Term and string analysis in goprofile (<https://biit.cs.ut.ee/gprofiler/gost)>and String (<https://string-db.org/)> (Supplementary Dataset 1).

**Reference**

1. Stebbins, M.J.*, et al.* Human pluripotent stem cell-derived brain pericyte-like cells induce blood-brain barrier properties. *Sci Adv* **5**, eaau7375 (2019).

2. Wang, L.*, et al.* Loss of NARS1 impairs progenitor proliferation in cortical brain organoids and leads to microcephaly. *Nat Commun* **11**, 4038 (2020).

3. Kadoshima, T.*, et al.* Self-organization of axial polarity, inside-out layer pattern, and species-specific progenitor dynamics in human ES cell-derived neocortex. *Proc Natl Acad Sci U S A* **110**, 20284-20289 (2013).

4. Susaki, E.A.*, et al.* Advanced CUBIC protocols for whole-brain and whole-body clearing and imaging. *Nat Protoc* **10**, 1709-1727 (2015).
